# Supplementary material for: What Is an Extreme Sports Healthcare Provider: An Auto-Ethnographic Study of the Development of an Extreme Sports Medicine Training Program
Source: Int J Environ Res Public Health. 2022 Jul 7;19(14):8286. doi: 10.3390/ijerph19148286 (PMC9323505; doi:10.3390/ijerph19148286)
Supplement: Supplementary file 1 [file ijerph-19-08286-s001.zip › ijerph-1724549-supplementary.pdf]

Supplementary Materials

Table S1. Results from the thematic analysis of transcripts.

Theme	Sub-theme	Summary of Typical Phrases
Learner factors	Person centered as well and skill based - Adaptable, multi-skilled and ecological	sound knowledge of personal capacities … underpinned by a sense of humility and discovery, rather than winning" - spoke to the qualities of the athlete, but also seemed to shout out that these could be the same qualities required for an effective extreme sports medicine practitioner.<br><br>no single no "fit" but rather a health practitioner (and certainly not necessarily a doctor) who can understand the environment, understand the sport and the equipment and technology that accompanies it, understand the athlete and be able to work pro-actively as well as reactively in a setting pertinent to that sport.<br><br>multiple skills/qualifications in order to be a more professional and employable ESM physician.<br><br>it is a rare expedition that can afford to pay wages to the accompanying doctor, unless that doctor has supplementary skills with a commercial focus.<br><br>appreciation for the sports, the environments and the athletes
	One-size does not fit all (multi-professionals/ multi-activities)	depends upon the role taken as an ESM Practitioner – that of supporting an endurance race in a remote setting such as the Marathon de Sable needs a different knowledge and skill set from one who provides support for a kayak crossing of the Tasman Sea, and different again from working in a medical clinic and seeing competition climbers with overuse injuries.<br><br>three different, but potentially overlapping types or functions – those provide clinic based services with an injury treatment and rehabilitation focus, those providing pre-event support and advice, and those providing either on scene "conventional" medical support at an event or remote "Telemedical" support to an event. These are all equally valid but quite different.<br><br>there won't be a single type of ESM health practitioner and we don't need that to enhance the health and performance of extreme sports athletes. What we need is practitioners, from multiple disciplines, who are interested and knowledgeable in a (or more than one) sport, with the skills to help the athlete or event organiser, in the way that they will be working.

		I have worked as Medical Director on events such as the Ultra Trail Australia, Big Red Run, Big Red Bash, Tough Mudder and Coast Trek. I have accompanied expeditions to Antarctica, the Arctic, the Himalaya, the Andes, the Karakorum, the far outback, the stratosphere and the deep ocean (Marianas Trench).
	Prepare for life-long learning	They will also have a template for learning about extreme sports that they can apply to endeavors that are not included in the course syllabus<br><br>No student, nor faculty member, nor ESM health practitioner will be an expert in all of the sports, so the course needed to allow students to do a "deep dive" (pun intended) in two or three sports but offer enough sports that every student could choose a diverse range of content to support their learning.
	Professional development	some students would have their own sporting pedigree and some would have no idea about an active and outdoors adventure, let alone a "mission" or a sporting event – extreme or otherwise. The course needs to meet the needs of both ends of the spectrum, but hopefully it inspires those that are less active to adventure into nature, not necessarily to be extreme but certainly be active and by extension, healthier.<br><br>greater than the sum of its part aspiration also extends to prescribing this type of activity to the patients that they encounter for the betterment of the health of the community more broadly.<br><br>team sport health practitioners may not be familiar with. An 'off-day' or 'choke' or 'error of judgement' will have different consequences for the ES athlete compared to the golfer, tennis player, cricketer or net-baller. Further, the athlete that thrives on the encounter with risk or the extreme, probably has motivations wildly at odds with the competitive team sport athlete.
Task factors	Design needs to fit the extreme sports context	an exercise in learning a lot about the topic, in refining and re thinking some of my pre-existing ideas and approaches<br><br>in some extreme sports, there was a real need to seek out the nuanced expertise that couldn't be gleaned from books or papers, if we wanted our students to have an authentic learning experience<br><br>with a focus and knowledge of performance and health is a niche area, that I have worked across. Some of those sports has included athletes and sports that may be considered extreme, but the devil is in the detail and the extremeness in the eye of the beholder!<br><br>The fact that the definition of extreme sport mandates that the practice of the sport must involve risk of severe injury or death establishes separate physical and psychological domains
	New and innovative	an innovative project

		Whilst there was some research and conference-based activity in the northern hemisphere, I couldn't find any evidence of any existing educational programs<br><br>manage risk, not just take it. Most are consummate professionals interested in using technology to explore the boundaries of human performance and the limits of our environment. So, the fact that prior to 2021, there were no Australian trained, extreme sport physicians appalled me. One only has to watch mega-events like the X-Games to realise that Australians are well represented in the world of international extreme sports. As both an athlete and medical practitioner, I recognised the need for an Australian based Extreme Sport Medicine program. While there are other societies, universities and textbooks offering ESM in other countries the field is still underdeveloped.<br><br>beyond the medical profession (as anticipated) to involve psychologists, engineers, coaches, nutritionists and athletes themselves, but even the backgrounds of those within medicine were very disparate: radiologist, intensivist, rehabilitation physician and orthopaedic surgeon for example, not just the sports physician or generalist primary and emergency care doctors
	Not just fixing things that have gone wrong but also pre-event work and during-event work (beyond the medical model)	proactive, preventative or performance space<br><br>able to work pro-actively as well as reactively in a setting pertinent to that sport<br><br>provide clinic based services with an injury treatment and rehabilitation focus, those providing pre-event support and advice, and those providing either on scene "conventional" medical support at an event or remote "Telemedical" support to an event<br><br>The ESM health practitioner is able to go beyond the question of 'why would you do that?' and into the territory of 'how can I help you manage the physical, emotional and psychological risk of that extreme activity?' The ESM health practitioner understands the physical risks of the sport and the psychological motivations of the athlete. The ESM health practitioner can offer non-judgemental advice on how to maximise physical preparation and flawless execution while staying within the bounds of 'acceptable risk". The ESM health practitioner can help an athlete foster and maintain psychological balance in extreme environments and extreme challenges. The ESM health practitioner can help identify and mitigate cognitive bias and unacceptable risk. The ESM health practitioner can be an objective, voice of reason more easily accepted and trusted by the ES athlete. Finally, the ESM health practitioner can be equipped with the most appropriate medication, gear and skills to provide treatment/stabilisation/evacuation to the injured ES athlete at events or individual challenges.<br><br>I witnessed more and more of my ES athlete colleagues get injured or die in circumstances that could only be described as 'human error', I realised the critical importance of understanding the motivations of ES athletes and the psychological pitfalls (such as cognitive biases) that can contribute to poor decision making and bad outcomes.

		My goal as an ESM health practitioner and ESM lecturer is to train health practitioners to recognise and mitigate physical and psychological risk in the extreme sport athlete cohort. My hope is that this will help lead to a reduction in morbidity and mortality in the extreme sports that are becoming exponentially more popular
Environment factors (social)	Educator needs to have passionate personal attributes, relevant professional background and learning innovators (experiential)	need to build some enthusiasm and contributions from a wide range of people (Educators).<br><br>extensive experience in critical care, expedition medicine and an unmatched pedigree in Australia of both providing medical support as well as participating to a high level in several extreme sport disciplines.<br><br>you need people who have the depth of clinical experience, a deep understanding of preferably several extreme sports and the interest in education, and somehow be able to employ and stimulate these “out of the box” non-conformist souls within a very “closed box” conformist University setting<br><br>passion for projects in this space or to call it their hobby.<br><br>I am enthusiastic about outdoor pursuits and I love big mountain backcountry skiing
